# An Analysis of How Smart Home Product Attributes Influence Older Adults’ Avoidance Psychology: The Sequential Mediation Role of Product Identity and Trust

**DOI:** 10.3390/bs14111060

**Published:** 2024-11-07

**Authors:** Yarong Huang, Xinxiang Li, Shuai Ling, Can Zheng

**Affiliations:** 1 Department of Industrial Design, Hongik University, Seoul 04066, Republic of Korea; huangyarong@g.hongik.ac.kr; 2 Department of Design and Manufacturing Engineering, Jeonbuk National University, Jeonju 54896, Republic of Korea; canzheng@jbnu.ac.kr; 3 College of Art and Design, Nanjing Forestry University, Nanjing 210037, China; shuailing@njfu.edu.cn

**Keywords:** smart home product, product attributes, product identity, trust, avoidance

## Abstract

As an effective method of improving the quality of life of older adults, smart home products have seen significant development and increased popularity in recent years. However, studies on the anti-consumption behaviors of older adults regarding these products remain relatively limited. Therefore, this study aims to investigate the avoidance behavior of older adults toward smart home products by investigating product attributes. The study proposes a theoretical model, “product attributes-product Identity-trust-avoidance behavior”, to explore the underlying mechanisms of avoidance behavior from both the psychological and the behavioral perspectives. Based on data from 506 valid questionnaires, the findings reveal that product attributes, product identity, and trust can significantly and negatively impact the avoidance behavior toward smart home products. In addition, product identity and trust play significant mediating and serial mediating roles between product attributes and smart home product avoidance behaviors. These findings provide valuable insights for smart home product manufacturers seeking to understand the avoidance behavior of older adults concerning their products. They also offer valuable guidance on design concepts, marketing strategies, and market formulation, providing new theoretical and practical recommendations for expanding the smart home market for older adults.

## 1. Introduction

Smart home systems incorporate health services and environmental assistive technologies, serving a pivotal role in improving the quality of life of older adults [1,2,3]. With the advancement of technology and social progress, the role of smart home products has become increasingly significant in enhancing the quality of life of older adults [4]. According to the WTO’s 2022 report, the number of individuals aged 65 and over is increasing at a faster rate than that of those under 65. Additionally, by 2050, the global number of people aged 65 and over is projected to rise from 10% in 2022 to 16%. At that time, the population of individuals aged 65 and over will be twice the number of children under 5 years old [5]. In 2019, the scale of China’s smart elderly care industry reached approximately CNY 3.2 trillion, with a compound annual growth rate exceeding a total of 18% over the past three years. By 2020, the industry is projected to reach a scale of CNY 4 trillion [6]. With the growing number of elderly consumers, the potential of the smart home product market has been further validated [7]. Interestingly, the relevant literature indicates that as of 2020, only 11.2% of internet users in China were aged 60 and above [6]. This may be due to the rapid decline in physical abilities among elderly consumers, significantly limiting their ability to use smart products. Additionally, elderly individuals often feel overwhelmed when faced with the complex functionalities of such products. Therefore, effectively addressing these challenges could greatly enhance the competitiveness of smart home products within the elderly consumer market.

Research indicates that while smart home products have achieved technological maturity, their adoption rate among older adults remains notably low. This observation can be attributed to the conservative attitudes of older adults toward new products and technologies. Technological innovations may disrupt established perceptions of existing products, contributing to resistance [2,3,8,9]. Smart home products can influence the daily lives of elderly individuals in a non-intrusive manner. This subtle integration allows these products to reduce elderly consumers’ resistance to technological changes [10]. This way, they can be guided more naturally toward gradually adapting to and accepting the technological innovations that new products introduce. On the other hand, modifying product attributes can also shape consumer evaluations of smart home products. For example, adding new attributes to a product can enhance consumers’ evaluations of the product. However, in highly complex products, the addition of new attributes may instead lead to lower consumer evaluations [11]. At the same time, existing research indicates that product attributes are a primary factor in consumer purchasing decisions. Furthermore, these attributes shape consumers’ attitudes toward smart products by generating varied consumer experiences [12,13]. However, as a multi-attribute product, smart home products exhibit varying effects on consumer attitudes depending on the specific combination of attributes [14,15]. Previous research has examined product attributes from various perspectives [12,14,16,17,18]. However, much of the existing research focuses on only one or two levels of intrinsic or extrinsic product attributes [14]. Existing research often overlooks the complex and multifaceted relationships between influencing variables. Additionally, there is a notable gap in studies focusing on product attributes specifically relevant to older adults. Consequently, it is imperative to investigate the effects of multi-attribute products of smart homes on this demographic.

Smart home products offer a broader array of solutions for managing complex home environments [1]. Scholars have proposed various solutions for smart home design from multiple perspectives to address the specific needs of older adults [19,20]. Nevertheless, older individuals continue to face several concerns and barriers when adopting smart home products, including issues related to reliability, trust, perceived burden on others, lack of perceived need, and cognitive load [21,22]. In particular, excessive information and cognitive overload can contribute to experiential avoidance [23,24], and, ultimately, affect consumers’ avoidance behavior [15]. A relationship exists between avoidance behavior and purchase intention, such that increased avoidance and hesitation correlate with a decrease in consumers’ purchase motivation [25]. Concurrently, relevant research indicates that product identity plays a crucial role in shaping consumer purchasing decisions. A strong product identity can lead to more targeted purchasing behavior, while a weak or unclear identity may result in consumer rejection or avoidance [26]. Researchers suggest that the positive impact of consumers’ willingness to repurchase or recommend products or brands they identify with is more pronounced. Additionally, such identity can attenuate negative emotions, such as post-purchase regret, experienced by consumers [27]. It is important to note that trust and avoidance are interrelated factors. Research indicates that when consumers have a high level of trust in an activity, their willingness to engage with it increases, compared to instances where trust is lacking. Moreover, even in the presence of inherent risks, trust in the activity tends to diminish avoidance behavior [28]. Therefore, based on the potential significance of consumers’ product identity and trust in smart home products in influencing avoidance behavior, this study integrates these factors into the research model.

Drawing on extensive prior research on smart homes, it is evident that existing studies predominantly emphasize smart home technology, platforms, and services. Most of this research broadly examines the impact of the overall product ecosystem on the relationship between older consumers and smart home products [1,20,29,30]. However, the influence of smart home product attributes on this relationship has been largely overlooked. Consequently, investigating the impact of these attributes on older consumers is essential for addressing their specific needs and enhancing their quality of life [31]. In the context of consumption psychology, technology acceptance, and usage habits, adolescents and middle-aged consumers can significantly differ from elderly consumers [32]. However, smart home manufacturers often overlook these differences in their market adaptation processes. Therefore, this study emphasizes the need for a more thorough investigation of the specific needs of elderly consumers. It also aims to create a relational model between elderly consumers and smart home products, clarifying the role of product attributes in this influence relationship.

This study aims to develop a model of smart home product avoidance from the perspective of older adults. Specifically, the paper has two primary objectives:

1. Explore the relationships in this influencing process.
2. Assess the extent to which the model offers enhanced theoretical and practical support for understanding smart home product avoidance.

In alignment with these objectives, this study seeks to address the following research questions.

1. What is the impact of product attributes on product identity, trust, and avoidance behavior concerning smart home products from the perspective of older adults?
2. How do product identity and trust impact the avoidance behavior of older adults with regard to smart home products?
3. Is there a mediating effect of product identity and trust within this model for older adults?

In response to these issues, this study established a research framework on how smart home product attributes affect consumers’ avoidance behavior. First, the relationship between the variables in the model was tested, and the potential chain mediation effect was explored, filling the research gap on the impact of smart home products on the avoidance behavior of older adults. This study provides valuable insights into the development of smart home products in this market, provides a unique research perspective on reducing the avoidance behavior of older adults toward smart home products, and further improves the research system of smart home products. This study is organized into seven separate sections. The Section 1 provides an overview of the current state of smart home products and outlines the primary issues faced by older adults. The Section 2 details the significant contributions and research hypotheses of the study and presents the research model. Section 3 and Section 4 are dedicated to the statistical methodologies and data analysis procedures that are employed in the study. Section 5 and Section 6 discuss the research findings and test the hypotheses, culminating in the drawing of conclusions. The Section 7 addresses the study’s limitations and proposes new directions for future research.

## 2. Research Hypothesis and Theoretical Framework

### 2.1. Theoretical Framework

#### 2.1.1. Product Attributes and Product Identity

According to the information processing theory, the information presented by a product constitutes the foundation upon which consumers build their understanding of the product [33]. Studies on the topic posit that the information conveyed by a product can be categorized into two distinct components: the intrinsic physical properties of the product and the external information derived from these physical properties. Consequently, product attributes are classified into two categories: tangible attributes, which encompass the product’s physical characteristics, and intangible attributes, which pertain to social dimensions associated with the product [14,15]. Furthermore, provided that tangible attributes of a product are the most readily perceptible to consumers, prior research has predominantly concentrated on examining the effects of these tangible attributes on consumer behavior [34]. However, product attributes encompass not only tangible attributes but also include intangible attributes [18]. Thus, focusing solely on these tangible attributes may constrain the consumers’ ability to engage in multidimensional trade-offs during the decision-making process [14]. Different product attributes exert varying effects on consumer satisfaction, and there exists an interdependent relationship among these attributes. For instance, once primary needs are fulfilled, supplementary product attributes can enhance consumer satisfaction further [35]. Therefore, consumers base their purchasing decisions on various product attributes, although not all attributes hold equal significance. Certain attributes are deemed more critical than others by consumers [36]. Research indicates that distinct product attributes are critical for addressing the needs of diverse consumer segments [37]. Additionally, relevant research suggests that consumers’ perceptions of product attributes encompass three primary components: the product itself, personal factors, and the social environment [38]. Additionally, the purchase motivations of elderly consumers can generally be divided into two categories. On one hand, their purchasing behavior aims to satisfy needs for belonging and social identification. On the other hand, it reflects self-oriented needs [39]. Therefore, this study identifies three categories of product attributes that may play a crucial role in elderly consumers’ evaluations of smart home products. The first one is “Similarity”. Similarity indicates the degree to which smart home products resemble traditional products in function, purpose, appearance, or other characteristics. The second one is “Social Influence”. Social influence indicates the impact of external factors such as others’ opinions, social norms, and cultural influences on decision making and use of smart home products. Finally, the third one is “Cognition”. Cognition describes the understanding and perception of products based on past experiences. Through these three approaches to product attributes, this study examines the avoidance relationship between elderly consumers and smart home products.

The concept of identity originates from the social identity theory, which posits that identity involves the perception of unity or belonging to a particular group. According to Jin Suk Park, social identity is characterized by the perceived similarity between individuals and social entities (such as organizations) and the strong emotional connection that binds them [40]. The concept of brand identity is derived from the social identity theory [41]. In addition, brand identity forges a connection between the brand and consumers, and enhances consumer preferences during the purchasing process [42]. In the context of brand identity, the level of brand identity exhibited by consumers varies across different product categories [43]. Consumers are more likely to purchase products from brands which they identify with, as they perceive that such purchases enable them to project their desired self-image [44]. The role of products, in this context, is significant [45]. Product identity is a process through which consumers establish a connection between their self-identity and the product, leading to modifications in their behavior and attitudes [46]. Building on the social identity theory and related research, this study defines product identity as the alignment between a product and an individual’s self-concept, informed by their prior experiences.

Related research shows that consumers’ perceptions of product attributes during the purchasing process are shaped by individuals in their social environment, especially those who share similar social status or image. This influence stems from consumers’ identification with these individuals, which, in turn, affects their evaluative judgments during the purchasing decision [47]. Additionally, studies indicate a significant link between product identity and product attributes. Specifically, product familiarity—representing consumers’ awareness and understanding of product attributes—is positively related to product identity. This implies that greater familiarity with a product enhances consumers’ identity with it [48,49]. Simultaneously, the impact of a product’s social influence on consumer behavior can be categorized into three levels: compliance, identity, and internalization. Consequently, during the product purchase process, social influence can affect consumer behavior, with the degree of consumer identity varying following the intensity of the social influence [50,51]. Research in the field of smartphones has demonstrated that consumers’ perceptions of smartphone attributes play a crucial role in shaping their identity with these devices [52]. Consequently, this study posits the following hypotheses regarding the relationship between product attributes and product identity.

**Hypothesis** **1a** **(H1a).** *Product similarity has a positive impact on older adults’ identities with smart home products.*

**Hypothesis** **1b** **(H1b).** *Product social influence positively influences older adults’ identities with smart home products.*

**Hypothesis** **1c** **(H1c).** *Product cognition positively influences older adults’ identities with smart home products.*

#### 2.1.2. Product Attributes and Trust

Trust refers to an individual’s belief or expectation regarding the reliability and ethical behavior of other individuals or entities, influenced by subjective factors such as confidence, risk, and safety [53]. Existing research indicates that consumers take into account the interactions between other consumers and organizations during their decision-making processes, using these interactions as reference points to predict their own behavior and anticipated outcomes. This process of behavioral inference is considered central to the concept of trust. In other words, consumers’ trust is primarily based on their observation and evaluation of the relationships between other consumers and organizations, which subsequently shapes their expectations regarding their own behavioral outcomes [54]. Moreover, compared to other age groups, elderly consumers generally place a greater emphasis on the importance of trust. They regard trust in others as the foundation for maintaining emotional connections [55]. This trust is not only present in their interpersonal relationships, but can also extend to their trust in products. When elderly consumers establish a sense of trust in a specific brand or product function, they are more likely to believe that the product can meet their actual needs. That way, they can experience a sense of security and belonging during its use. Therefore, trust plays a critical role in examining the relationship between elderly consumers and products. Trust is a multidimensional social construct that evolves in response to shifting contextual factors [56,57]. It involves the consumer’s evaluation of the reliability and dependability of an individual or entity. Consumers’ trust in products derives from their confidence in the product’s quality and reliability [58]. Therefore, this study conceptualizes their trust in smart home products as encompassing their confidence in the product’s quality, security, and experiential reliability.

The existing literature indicates that both the tangible and intangible attributes of a product significantly influence consumers’ trust in the product [59]. Moreover, trust can be enhanced through direct experience and various other methods. Empirical studies have demonstrated that positive experiences such as those encountered by international students in a host country can significantly enhance their trust in that country [60]. William H. Dutton’s research further substantiates this relationship [61]. Consumer experience can be categorized into direct and indirect types. Although direct experience generally exerts a more profound influence on subsequent consumer behaviors and attitudes, the impact of indirect experience on consumer behavior remains a significant area of interest [62,63]. Indirect experience encompasses the information consumers gather from a variety of sources—self-reported experiences, second-party inputs, and third-party sources—before engaging in the actual experience. This information includes past experiences, social influences, and evaluations from individuals within their social circles [63]. Much of the information obtained through indirect experience tends to be biased, leading consumers to focus disproportionately on a single product attribute while neglecting other significant attributes [62]. Direct experience with products can be categorized into two types: previous experience and current experience. Both types of direct experience are closely connected to consumers’ attitudes and behaviors [64]. Construal level theory further explains this by suggesting that as the psychological distance between individuals and an event or object increases, their perception of it becomes increasingly abstract [65]. When evaluating product similarity based on past experiences, consumers perceive that the degree of similarity can influence their attitude toward the product [66]. At the same time, consumers’ familiarity with products and brands can significantly influence their trust. High levels of familiarity with products tend to enhance consumers’ trust in them or their brands, and impact their purchase intentions [67]. Consumers’ trust in autonomous vehicles is more significantly shaped by social influences such as media coverage and peer factors than by their perceptions [68]. It has also been confirmed that social influence can significantly impact trust. For instance, in social networking services (SNSs), users’ negative evaluations can affect trust in the community service [53]. Relevant research indicates that even though 3-year-old children may struggle to differentiate information sources, they still rely on their intellectual ability to discern information. Moreover, the more they know, the greater their trust in that information [69]. Therefore, this study proposes the following hypotheses regarding product attributes and trust.

**Hypothesis** **2a** **(H2a).** *Product similarity has a positive effect on the trust of older adults in smart home products.*

**Hypothesis** **2b** **(H2b).** *Product social influence can positively influence the trust of older adults in smart home products.*

**Hypothesis** **2c** **(H2c).** *Product cognition can positively influence older adults’ trust in smart home products.*

#### 2.1.3. Product Attributes and Avoidance Behavior

Avoidance refers to a behavior in which consumers actively seek to reduce their exposure to a particular substance [70]. Related research also supports this view, suggesting that avoidance is a phenomenon where consumers deliberately distance themselves from or reject certain brands [71]. ÉK Vajkai posits that avoidance behaviors arise when consumers’ expectations for products are not met [72]. Thus, for consumers, product avoidance behavior manifests as a deliberate act of rejection or avoidance when a product fails to meet their expectations.

Elderly consumers may encounter certain adaptation barriers when facing smart home products, particularly as their physical and cognitive abilities decline. These barriers can impose constraints on their consumption behavior [73]. This could make them more susceptible to developing avoidance tendencies or experiencing other negative emotions when using smart home products. Meanwhile, positive cognitive evaluations and emotional attitudes toward product attributes or product-related information are likely to mitigate consumers’ avoidance behaviors [15]. Research indicates that consumers’ familiarity with product attributes can significantly influence their avoidance behavior. Moreover, literature from related fields posits that negative social influence is strongly associated with avoidance behaviors [74]. For instance, consumers’ social evaluations of individuals or entities can significantly influence their avoidance behavior [75]. The association between cognition and avoidance has been empirically established. For instance, individuals with higher levels of experiential avoidance may experience greater discomfort and negative emotions when watching high-emotion films, with cognitive demands moderating this relationship [76]. The attributes of smart home products can significantly influence consumer avoidance behavior. Empirical research indicates that incorporating distinctive product attributes can mitigate such avoidance behavior [15]. Consequently, this study combines findings from relevant research domains and formulates the following hypotheses concerning the relationship between product attributes and avoidance behavior.

**Hypothesis** **3a** **(H3a).** *Product similarity exerts a negative effect on the avoidance behavior of older adults toward smart home products.*

**Hypothesis** **3b** **(H3b).** *Product social influence can exert a negative impact on older adults’ avoidance behavior toward smart home products.*

**Hypothesis** **3c** **(H3c).** *Product cognition can exert a negative impact on older adults’ avoidance behavior toward smart home products.*

#### 2.1.4. Product Identity and Trust

Research indicates that consumers’ identity with a product or brand can significantly influence their trust in that product or brand [77]. Relevant studies have confirmed that the development of identity is influenced by the regulation of trust [78], with a higher degree of product identity being associated with stronger trust. When consumers identify with a person or brand, their trust in the products recommended by that person or brand is significantly enhanced [79]. This effect is particularly strong when consumers have used related products, as their perceptions of product reliability and recognition become more pronounced. This enhanced recognition can significantly influence their trust in the products [80]. Therefore, this study proposes the following hypothesis concerning the interplay between product identity and trust.

**Hypothesis** **4** **(H4).** *Product identity can positively influence the trust of older adults in smart home products.*

#### 2.1.5. Trust and Avoidance Behavior

Relevant studies have confirmed that increasing interpersonal trust can mitigate social avoidance in the treatment of depression [81]. When companies collaborate with countries characterized by high social trust, their avoidance of uncertainty is reduced. Conversely, collaboration with countries that exhibit low social trust prompts companies to assess cooperation risks more critically, leading to increased avoidance behavior [82]. Consumers’ trust in a company can significantly influence their psychological sense of security. When consumers experience a trust crisis regarding a specific travel brand or destination, it may trigger uncertainty and anxiety related to the travel process. This can potentially weaken their confidence in subsequent consumption decisions and affect their behavioral intentions and actual choices [83]. Similarly, a comparable phenomenon is observed in the context of brand trust: when consumers’ trust in a particular brand diminishes, their sense of identification and emotional attachment to that brand can also significantly decline, ultimately exerting a suppressive effect on their consumption behavior [84]. While existing research has not directly established a relationship between trust in products and avoidance behavior toward smart home products among older adults, this study posits that such a relationship may exist. Consequently, the study proposes the following hypothesis concerning the impact of trust on avoidance behavior.

**Hypothesis** **5** **(H5).** *Trust can negatively influence the avoidance behavior of older adults toward smart home products.*

#### 2.1.6. Product Identity and Avoidance Behavior

Research on the topic indicates a relationship between brand identity and brand avoidance. As products are integral to the evaluation framework of brand identity, consumers’ identification with products is also influenced by this relationship [85]. Moreover, by establishing a connection between celebrities and products, companies can effectively transfer consumers’ identification with the celebrity to the product. This way, they can enhance their recognition of the product. This association helps to mitigate consumers’ resistance to advertising messages and reduces their tendency to avoid marketing information [86]. Additionally, relevant research suggests that the “approach-avoidance” relationship better captures the dynamics between consumers and brands or products than other conceptual frameworks [87]. Consequently, this study proposes the following hypothesis regarding the relationship between product identity and avoidance behavior.

**Hypothesis** **6** **(H6).** *Product identity can negatively influence the avoidance behavior of older adults toward smart home products.*

#### 2.1.7. The Mediation Effect of Trust and Product Identity in Avoidance Behavior

Based on the aforementioned assumptions, this study posits that product identity and trust mediate the relationship between older adults’ avoidance behavior and smart home products. Mitigating resistance to smart home technology adoption can be achieved by influencing avoidance behaviors through the modification of product attributes. This reduction in resistance strengthens older adults’ identification with smart home products and promotes a subjective belief in their supportive role in daily life [49,50,51,52,76,88]. This cognition can bolster older adults’ trust in smart home products (Hypothesis H5) [77]. Consequently, enhancing their identity with and trust in smart home products through targeted product attributes may reduce their avoidance behavior toward these products. Based on this premise, this study proposes the following intermediary hypotheses concerning product identity and trust.

**Hypothesis** **7a** **(H7a).** *Product identity can serve as a mediator in the relationship between product similarity and the avoidance behavior of older adults toward smart home products.*

**Hypothesis** **7b** **(H7b).** *Product identity can act as a mediator between the social impact of products and the avoidance behavior of older adults toward smart home products.*

**Hypothesis** **7c** **(H7c).** *Product identity can serve as a mediator between product cognition and the avoidance behavior of older adults toward smart home products.*

**Hypothesis** **7d** **(H7d).** *Trust can serve as a mediator between product similarity and the avoidance behavior of older adults toward smart home products.*

**Hypothesis** **7e** **(H7e).** *Trust can serve as an intermediary between the social impact of products and the avoidance behavior of older adults toward smart home products.*

**Hypothesis** **7f** **(H7f).** *Trust can serve as an intermediary between product cognition and the avoidance behavior of older adults toward smart home products.*

**Hypothesis** **7g** **(H7g).** *Product identity and trust can have a serial mediating effect between product similarity and the avoidance behavior of older adults toward smart home products.*

**Hypothesis** **7h** **(H7h).** *Product identity and trust can have a serial mediating effect between the social influence of products and the avoidance behavior of older adults toward smart home products.*

**Hypothesis** **7i** **(H7i).** *Product identity and trust can have a serial mediating effect between product cognition and the avoidance behavior of older adults toward smart home products.*

### 2.2. Research Hypothesis

**Figure 1:**
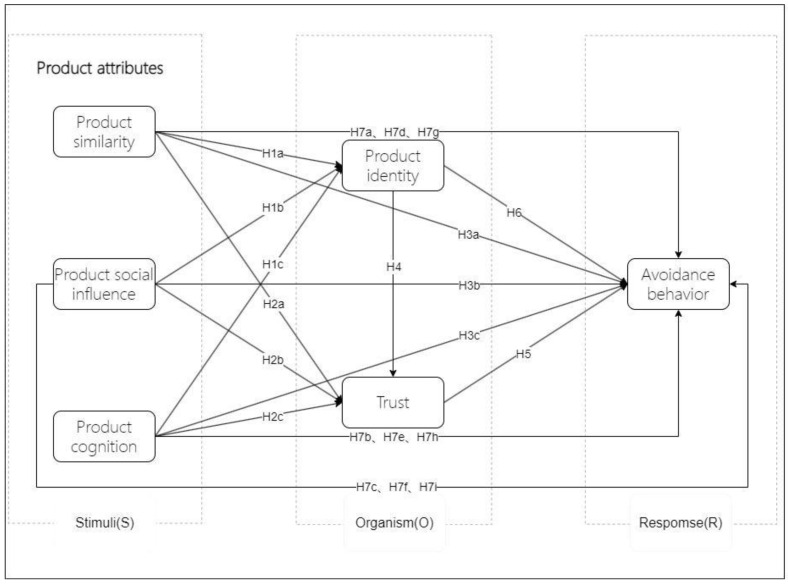
Conceptual model of this study.

The S-O-R (stimulus–organism–response) theory is extensively utilized in examining the interactions between consumers and products [89,90,91]. This theory posits that “S” denotes external stimuli, “O” signifies internal states shaped by these stimuli, and “R” reflects the behavioral responses elicited by the stimuli [90,91,92]. This study designates product similarity, product social impact, and product cognition as the stimulus factors (“S”), while product identity and trust are categorized as the organic factors (“O”), building upon established research models. Avoidance is considered the behavioral response (“R”). Based on these classifications and the SOR theory, the research model for this study is subsequently developed, as illustrated in Figure 1.

## 3. Methodology

### 3.1. Background and Questionnaire Survey

According to the 2023 China Smart Home Development Report, the demand for smart home products in China continues to increase. This growth is fueled by an increased emphasis on improving home life quality and user experience, especially in the aftermath of the pandemic. It is projected that by 2025, the size of China’s smart home market will reach CNY 952.3 billion, representing a year-on-year growth rate of 28.4% [93]. Consequently, this study was conducted with a focus on China.

The questionnaire project of this study was adapted and designed based on the questionnaires used in other relevant studies. To ensure the reliability of the questionnaire, we initially recruited 6 relevant field experts in China to discuss the questionnaire. After localizing the questionnaire based on these discussions, we conducted a pilot survey with 36 elderly participants. We then optimized and refined the descriptive language of the experimental questionnaire through further discussion. This way, we managed to enhance its clarity and comprehensibility for respondents. Subsequently, the questionnaire scales for the six variables outlined above were extracted, as detailed in Table 1. All variables were assessed using a 7-point Likert scale, with responses ranging from 1 (strongly disagree) to 7 (strongly agree).

This study employs a quasi-experimental research design, which minimizes the in-fluence of confounding variables. This is achieved by carefully planning the questionnaire content and administration methods. Furthermore, data collection was conducted using a questionnaire survey approach. The questionnaires were distributed through both online and offline methods. At the same time, an incentive mechanism was implemented to voluntary participants, offering a reward of CNY 3 for each completed questionnaire. By permitting participants to voluntarily engage with the study, greater interest and motivation were fostered, resulting in more attentive and considered responses to the questionnaire. Consequently, the data collected are anticipated to more accurately reflect the participants’ true thoughts and attitudes.

### 3.2. Data Collection and Analysis Methods

This study employed a mixed-method approach for data collection, utilizing both online and offline questionnaires to address the challenges associated with the accuracy of online surveys among older adult participants. To ensure the reliability of the responses, the study incorporated integrity questions repeating identical questions at the beginning and end of the questionnaire to identify and exclude inconsistent responses. After the completion of the offline questionnaire survey, a brief follow-up interview was conducted. A selection of questions was randomly reasked to evaluate the consistency of respondents’ replies. The questionnaires that exhibited inconsistencies were subsequently discarded. Additionally, the content and format of both online and offline questionnaires were standardized and unified to ensure the consistency and comparability of the data. This approach reduces errors arising from respondents’ comprehension biases or response biases, thereby enhancing the validity of the research findings. Data collection began in June 2024 in China, resulting in a total of 626 completed questionnaires. After excluding 120 unreliable responses, 506 questionnaires were retained for structural equation modeling. This final sample size of 506 surpasses the minimum requirement of 10 times the number of measurement items (24 items), ensuring a robust data analysis [100].

SmartPLS can comprehensively assess the validity and rationality of all paths within the model and is capable of handling complex models. Therefore, it can elucidate the interrelationships among variables. It can also scientifically interpret the mechanisms of action within the model. This is achieved through the influence paths of latent variables, and the availability of reasonable predictions of causal relationships among relevant variables. This ensures the robustness and scientific rigor of the model. Accordingly, this study employed both SPSS27 (IBM) and SmartPLS4 (SmartPLS Executable) for data processing and analysis. SPSS27 was used initially to analyze the demographic characteristics of the respondents. Subsequently, SmartPLS4 was employed to assess key metrics including the external loadings, Cronbach’s alpha, composite reliability (CR), and average variance extracted (AVE). The research hypotheses were tested using the bootstrapping algorithm, and model fit was evaluated through R^2^ and Q^2^ values.

### 3.3. Respondent Demographic Characteristics

The demographic characteristics of the research respondents are shown in Table 2. The sample included 246 male-identifying participants (48.62%) and 260 female-identifying participants (51.38%). The age distribution is as follows: A total of 31.62% of the respondents were aged 55–60 years, while 35.97% were aged 61–65 years. These numbers are consistent with the demographic distribution of middle-aged and older adult populations reported by the China National Bureau of Statistics [101]. Additionally, the percentages of the respondents with educational backgrounds at high school level and below high school level were 31.62% and 46.05%, respectively, totaling 77.67% of the survey sample. This distribution is comparable to the current educational attainment levels reported for the older adult population [102]. The overall percentage of the respondents with a monthly income below CNY 4000 was 88.14%, which closely aligns with the reported average monthly income of CNY 3863 for the older adult population [103]. Therefore, the sample selected in this study exhibits a degree of representativeness, and the collected data are deemed suitable for use in subsequent research.

## 4. Results

### 4.1. Reliability and Validity Analysis

The reliability and validity test results are presented in Table 3. Cronbach’s alpha values range from 0.862 to 0.882, all exceeding the reference threshold of 0.7 [104]. The CR values range from 0.867 to 0.882, all of which exceed the reference value of 0.6 [105]. This suggests that the scale exhibits strong overall internal consistency, thereby ensuring its reliability. PLS-SEM employs external loadings and AVE as indicators to measure convergent validity, with external loadings ranging from 0.751 to 0.826, all exceeding the reference value of 0.6 [96]. The AVE values range from 0.620 to 0.652, all of which exceed the reference value of 0.5 [96]. This indicates that the scale has high convergent validity.

### 4.2. Discriminant Validity Analysis

This article employs two methods to assess the discriminant validity of the model, to better assess the reliability and validity of the model. The first method is the Fornell–Larcker criterion, which stipulates that the correlation coefficient between latent variables should be less than the square root of their respective AVEs. The second method is the HTMT (Heterotrait–Monotrait Ratio), where the HTMT value should be less than 0.85 [105]. In this study, the HTMT values for all variables were below 0.85, indicating that significant deviation was not present. Furthermore, this result further confirms that the reliability and validity of the research are at a high level. The results are presented in Table 4 and Table 5.

### 4.3. Collinearity Analysis

To verify the results of the structural equation model, it is essential to first determine whether the collinearity diagnosis VIF value is less than 5 [96]. When the VIF values of the data are below 5, then there is no severe multicollinearity effect, and the model coefficients are unlikely to experience significant bias issues. The results of this study, as presented in the following table, show that the VIF values range from 1.267 to 1.473, indicating the absence of severe multicollinearity and robustness issues. As demonstrated in Table 6.

### 4.4. Path Analysis

**Figure 2:**
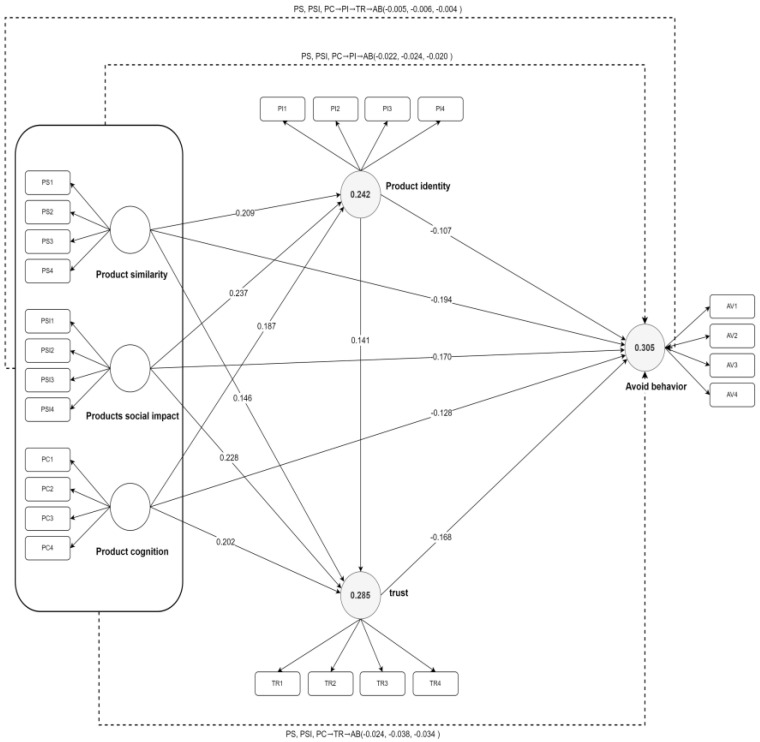
Analytical results of the model.

Using SmartPLS4 to test the path coefficients of the structural model, the path coefficient “t” value must be greater than 1.96 to be considered significant [104]. The path coefficient has passed the test at a level of significance of 5%, indicating that the path coefficient is significant. The analysis results are shown in Table 7 and Figure 2. It can be concluded that PS (β = 0.209, T = 4.812, *p* = 0.000), PSI (β = 0.237, T = 5.450, *p* = 0.000), and PC (β = 0.187, T = 4.261, *p* = 0.000) have significant positive effects on PI. Therefore, hypotheses H1a, H1b, and H1c are valid. Meanwhile, PS (β = 0.146, T = 3.330, *p* = 0.001), PSI (β = 0.228, T = 4.864, *p* = 0.000), and PC (β = 0.202, T = 4.522, *p* = 0.000) have significant positive effects on TR. Therefore, hypotheses H2a, H2b, and H2c are true. In addition, PS (β = −0.194, T = 4.060, *p* = 0.000), PSI (β = −0.170, T = 3.738, *p* = 0.000), and PC (β = −0.128, T = 2.774, *p* = 0.006) have significant negative effects on AB; thus, it is assumed that H3a, H3b, and H3c are valid. PI (β = 0.141, T = 3.248, *p* = 0.001) has a significant positive effect on TR; thus, it is assumed that H4 is true. TR (β = −0.168, T = 3.681, *p* = 0.000) and PI (β = −0.107, T = 2.374, *p* = 0.018) have a significant negative impact on AB; therefore, hypotheses H5 and H6 are true. In addition, AB plays a mediating role between PS and PI (β = −0.022, T = 2.020, *p* = 0.043), PSI and PI (β = −0.025, T = 2.177, *p* = 0.029), and PC and PI (β = −0.020, T = 2.048, *p* = 0.041). Therefore, it is assumed that H7a, H7b, and H7c are true. AB plays a mediating role between PS and TR (β = −0.024, T = 2.475, *p* = 0.013), PSI and TR (β = −0.038, T = 2.931, *p* = 0.003), and PC and TR (β = −0.034, T = 2.731, *p* = 0.006). Therefore, it is assumed that H7d, H7e, and H7f are true. PI and TR play a sequence mediating role between PS and AB (β = −0.005, T = 2.185, *p* = 0.029), PI and TR between PSI and AB (β = −0.006, T = 2.179, *p* = 0.029), and PI and TR between PC and AB (β = −0.004, T = 2.086, *p* = 0.037). Therefore, it is assumed that H7g, H7h, and H7i are valid.

### 4.5. Explanatory Power and Predictive Power of the Model

R^2^ represents the overall impact of all predictor variables on latent variables, while Q^2^ is used to evaluate the predictive ability of the model. When the R^2^ value is greater than 0.20 (with the R^2^ values of PS, PSI, and PC all exceeding 0.20) [104,106], this indicates that the model demonstrates strong explanatory power. When the Q^2^ value is greater than 0 (with the Q^2^ values of PS, PSI, and PC all exceeding 0) [107], this indicates that the model possesses strong predictive capability, as demonstrated by the results presented in the following Table 8.

## 5. Discussion

This study explores the impact of smart home product attribute evaluation (including at the product level, social level, and individual level) on avoidance behavior in smart home products. It also analyzes the sequential mediating role of product identity and trust in this relationship.

First of all, product similarity, social impact, and product cognition in product attributes all have a negative impact on avoidance behavior, which is similar to the results of some relevant studies [48,49,51,52]. A notable feature of this study is its multidimensional approach to assessing product attributes. Unlike prior research that has typically focused on individual aspects of product attributes, this study incorporates considerations of product similarity, social impact, and cognition to investigate their collective influence on avoidance behavior. The data indicate that, compared to social impact and cognitive evaluations of products, product similarity exerts a more significant influence on consumers’ avoidance behavior. Specifically, the findings suggest that older adults’ avoidance behavior toward smart home products is more strongly affected by product familiarity than by social evaluations or individual cognition. This suggests that greater familiarity with products achieved by incorporating elements of traditional product appearance and usage methods can significantly reduce avoidance behavior among older consumers. Therefore, designing smart home products with familiar features from traditional products can effectively mitigate resistance and facilitate acceptance among older adults.

Furthermore, from the research results, it can be seen that product similarity, product social impact, and product cognition all have a positive impact on product identity. Although relevant studies have shown that social impact has the same effect on product identity as in the results of this study [46], the influence of other potential factors on product identity has not been thoroughly explored. Additionally, while the majority of the existing literature primarily examines the relationship between brand identity and products, this study shifts the focus to investigating how product attributes affect product identity. By doing so, it broadens the scope of research on product attributes and product identity and extends the concept of product identity into the context of smart home product avoidance. At the same time, product cognition has relatively little influence on the avoidance behavior of elderly consumers, especially in comparison with product identity, since it primarily relies on their subjective judgment, which is often insufficient due to limited information sources. In contrast, product social influence offers a more diverse array of information channels and reference groups. It enables elderly consumers to make judgments through multiple external information sources, thereby resulting in a more significant influence on product identity.

In addition, product identity and trust can significantly and negatively affect the avoidance behavior of older adults toward smart home products. This phenomenon can be understood through social identity theory and trust behavior theory. Specifically, when older adults cultivate a strong sense of identity with smart home products, they are more likely to adopt these technologies, which reduces their tendency to avoid them. Similar findings have been reported by researchers, such as Lu Lin, in the context of brand identity [108]. Additionally, consumers’ trust in a product significantly influences their purchasing or selection behavior. This finding aligns with existing research, which has similarly demonstrated the impact of trust on consumer decision making [70,81,82].

Furthermore, the present study found that product identity can positively influence trust, a finding that is consistent with prior research [80]. Unlike previous studies that extensively explored the relationship between product recognition and trust, this study investigates product identity in depth to elucidate the relationship between consumer trust and products. The research data reveal that, compared to the influence of product attributes, the impact of product identity on trust is relatively minor. This highlights the critical role of product attributes in building and maintaining consumer trust. Additionally, the explanatory power of models concerning product identity is lower compared to trust and avoidance. This may be attributed to the non-inclusion of the forgetting curve as an external factor. According to Ebbinghau’s forgetting curve theory, memory accuracy declines significantly over time [109]. Therefore, variations in the distance and experiences of the surveyed individuals may lead to differences in their evaluations of the product, which could impact the model’s fit to some extent. Nevertheless, the results of this study show that product identity remains a significant factor within the research model. This finding underscores the importance of product identity in understanding consumer behavior.

In addition, product identity and trust mediate the relationship between product attributes and avoidance behavior. This finding extends the application of product identity and trust as mediating variables within the model. It shows that consumers’ understanding of product attributes enhances their identity with and trust in the product, ultimately reducing their avoidance behavior. These results align with similar findings in relevant research topics [48,49,59]. This validates the significance of product identity and trust in influencing older individuals’ avoidance of smart home products. It also expands the theoretical research on anti-consumption behavior concerning smart home products, particularly under the lens of product identity.

Finally, the results reveal a sequential mediating effect of product identity and trust between product attributes and avoidance behavior. This finding contributes to the enhancement of theoretical models that focus on the consumption behavior of older individuals toward smart home products [30,110]. A similar analysis shows that older adults’ level of understanding of smart home products is closely related to their recognition of these products. Higher product recognition enhances consumers’ trust in smart home products and strengthens the connection between consumers and the products. Additionally, greater trust is effective in reducing avoidance behavior toward smart home products, significantly impacting consumers’ attitudes and behaviors. This finding highlights the causal sequence among product attributes, product identity, trust, and avoidance behavior, and underscores the critical role of a deep understanding of products in influencing older adult consumers’ responses.

The model in this study holds significant practical implications for the sustainable growth of the smart home industry. Essentially, this study highlights the important connections between product attributes, product identity, trust, and consumer avoidance, by examining the avoidance behavior of older consumers toward smart home products; this insight enables smart home companies to tailor their marketing strategies and product offerings to better address these factors, thereby enhancing product adoption among older adult users. However, we found that, although the sequential mediating effect is significant, the path coefficients are relatively small. This may be attributed to the fact that during the use of smart home products, elderly individuals may experience cognitive bias toward product functions or operations due to external influence originating from family members or friends [111]. Mainly due to reliance on assistance from others, elderly consumers may develop misconceptions about the complexity or usability of the product, which can subsequently affect their autonomous judgment and overall product usage experience.

## 6. Conclusions

This research model can forecast older adults’ avoidance behavior toward smart home products, offering both theoretical and managerial significance. Firstly, it validates the relationships between product attributes, product identity, trust, and the avoidance behavior of older adults toward smart home technologies.

Moreover, through causal sequence relationships, the impact mechanism of the interaction between product attributes, product identity, and trust on avoidance behavior was revealed, deepening the understanding of avoidance behavior among older adults in the context of smart home products. While previous studies have predominantly explored product identity from the designer’s perspective [46], the present study focuses on the consumer’s viewpoint. It aims to conduct an in-depth analysis of the relationship between product attributes and consumer product identity, thereby providing a more comprehensive understanding of how consumers develop a sense of identity during their product experiences. This study addresses the gap in research concerning the relationship between consumers and product identity in the context of smart home products, thereby enriching the theoretical concepts related to product identity. Additionally, it provides several theoretical insights.

First of all, the literature indicates that elderly consumer avoidance behavior is influenced by multiple factors [70,71,85,99]. This study identifies product similarity, social influence, and product cognition as key antecedent variables that can influence consumer avoidance behavior. Based on the social identity theory, it conceptualizes consumer trust and product identity as mediating variables. This way, it establishes a transmission mechanism for avoidance behavior. Additionally, consumer avoidance behavior is treated as the outcome variable of anti-consumption behavior. This study also proposes a comprehensive model of anti-consumption behavior that integrates the aforementioned theories and variables, through multivariable analytical methods. Essentially, it systematically reveals the underlying mechanisms that influence consumer avoidance behavior.

Additionally, this study introduces a novel research perspective on the subject by employing a multidimensional analysis of product attributes. It underscores the critical role that product similarity, social impact, and product cognition play in mitigating consumer anti-consumption behavior. A deep understanding of these attributes can effectively reduce consumers’ avoidance tendencies and is one of the important strategies to improve consumers’ product attitudes.

Ultimately, for the widespread adoption of smart home products within the older adult market, it is crucial to clearly articulate their benefits to this demographic [112]. Therefore, developing a systematic promotion strategy for smart home products is essential. Based on the research findings, we recommend the following strategies to expand the market for smart home products among older adults.

In designing smart home products, it is essential to balance functionality and design innovation with the consideration of traditional product forms and established consumer usage habits. This approach ensures that consumers can more readily adapt to these products and integrate them into their daily lives [48,49]. At the same time, the establishment of online and offline smart home product experience centers can attract consumers to actively participate and cultivate their habits of using the products. The design of online experience centers should focus on content quality and innovation, which can help enhance the social evaluation of smart home products and expand their influence. The design of offline experience centers should focus on the convenience and quality of the experience, establish a connection between smart home products and consumers, and increase consumers’ understanding of smart home products. These measures can enhance consumers’ trust in smart home products. Furthermore, the existence of such experience centers helps consumers establish habits and awareness of using these products.

On top of that, it is imperative to intensify the promotion and dissemination of smart home products. Industry professionals should employ a variety of methods to inform consumers about the advantages of smart home technologies and their distinctions from traditional products, thereby enhancing consumer awareness and understanding. In executing promotional strategies, emphasis should be placed on the diversification of promotional channels and the technical accuracy of the content. It is equally important to account for the unique characteristics of the smart home sector. These considerations are critical in determining the overall effectiveness of promotional campaigns.

Moreover, the government should incorporate the use of smart home products into smart aging policies, integrating health care and elderly care services. Equally, it should encourage nursing homes and community elderly care centers to adopt these technologies more widely, to enhance the quality of life and safety of older adults [113]. Additionally, it is essential to support local governments in implementing smart health and aging demonstration projects that showcase the advantages of smart home products through practical case studies. This approach can enhance the sentiments of acceptance and trust of older consumers. This policy combination has the potential to facilitate the widespread adoption and application of smart home technologies.

To conclude, it is crucial to enhance the after-sales service for smart home products to ensure a superior user experience. To achieve this, it is recommended to establish a dedicated and professional after-sales service team. This team should be responsible for conducting regular follow-ups and maintenance for smart home product users. That said, it is equally important to optimize the after-sales service process by simplifying the repair reporting system and implementing an efficient feedback mechanism. Such improvements reassure consumers that any issues with smart home products can be effectively resolved, thereby increasing their trust in and expectations of these products and enhancing user loyalty.

## 7. Limitations and Future Research

This study, while offering valuable insights that provide avenues for future research, includes several limitations. In particular, although avoidance behavior is a critical indicator of anti-consumption, it does not comprehensively capture actual purchasing behaviors. To address this, future research could employ observational methods or longitudinal studies to better understand the underlying mechanisms of consumer avoidance behavior. Furthermore, the participants in this study were exclusively older users from China, and consumer behaviors may vary across different regions. This geographical limitation could impact the applicability and generalizability of the research findings. Therefore, future studies should aim to collect data from diverse regions to ensure representativeness in terms of geographical areas, social contexts, and levels of technological experience, thereby further validating the effectiveness of the proposed model. In addition, the concept of smart home products is broad, and this study did not conduct an in-depth analysis of specific product categories. For instance, the frequency of use and the level of sophistication may vary among different types of smart home products, which could overlook some potential influencing factors. Future research should focus on detailed categorization studies of various types of smart home products. What is more, this study’s experiments primarily relied on surveys of older adults’ past experiences and did not sufficiently explore the psychological mechanisms involved in older adults’ acceptance of new technologies, nor the complex interactions with other influencing factors. Therefore, future studies should incorporate a broader range of influencing variables, particularly those related to the cognitive, emotional, and behavioral responses that may arise during older adults’ technology adoption process. Consequently, this will help to further elucidate the relationship between older adults’ acceptance and avoidance behaviors with regard to new technologies.

## Figures and Tables

**Table 1:** Measurement.

Constructs	Items	Sources
Product similarity	The similarity between this smart home product and traditional products in appearance.	Gianfranco Walsh [94]
	The similarity between this smart home product and traditional products in usage methods.	
	The similarity between this smart home product and traditional products in user experience.	
	This smart home product evokes a sense of familiarity for me.	
Product social influence	The usage of smart home products is influenced by the opinions of those around me.	Xiang Wang [6]
	People who use smart homes will appear more efficient than those who do not use them.	
	The use of smart home products is a trend.	
	Using smart home products can enhance personal social image.	
Product cognition	Using smart home products provides greater added value.	Yaoming Liang [95]
	Smart home products are simpler to use and operate.	
	Smart home products can help me better handle the inconvenience in my daily life.	
	Using smart homes can improve the quality of life.	
Product identity	I think smart home products are very easy to use.	Jin Suk Park [96]
	I think the operation of smart home products is in line with my habits.	
	I have a strong sense of belonging to smart home products.	
	When someone praises smart home products, I feel very happy.	
Trust	I don’t think using smart homes will lower my quality of life.	Earth Chandrruangphen, [97] Peter Wehnert [98]
	I think smart home products are very safe to use.	
	The user experience of this smart home will be as good as I expected.	
	I trust smart home products.	
Avoidance behavior	I don’t know how to use smart home products.	Michael S.W. Lee [99]
	I won’t establish any connection with smart home products.	
	When I need the relevant functions of smart home products, I will choose other products to replace smart home products.	
	I would rather choose traditional products than use smart home products.	

**Table 2:** Respondent demographic characteristics (N = 506).

	Item	Frequency	Proportion
Gender	Male	246	48.62%
	Female	260	51.38%
Age (in years)	55–60	160	31.62%
	61–65	182	35.97%
	66–70	100	19.76%
	>70	64	12.65%
Education	Below high school	233	46.05%
	High school	160	31.62%
	Junior college	75	14.82%
	Undergraduate	22	4.35%
	Postgraduate	16	3.16%
Monthly income (CNY/Yuan)	<3000	241	47.63%
	3000–4000	205	40.51%
	4000–5000	42	8.30%
	>5000	18	3.56%

**Table 3:** Reliability and validity analysis.

Construct	Item	Factor Loadings	Cronbach’s Alpha	CR	AVE
Product similarity (PS)	PS1	0.783	0.882	0.882	0.652
	PS2	0.815			
	PS3	0.804			
	PS4	0.826			
Product social impact (PSI)	SIP1	0.782	0.874	0.868	0.621
	SIP2	0.774			
	SIP3	0.807			
	SIP4	0.788			
Product cognition (PC)	PC1	0.794	0.865	0.867	0.620
	PC2	0.751			
	PC3	0.821			
	PC4	0.783			
Product identity (PI)	PI1	0.790	0.862	0.873	0.631
	PI2	0.792			
	PI3	0.774			
	PI4	0.821			
Trust (TR)	TR1	0.777	0.870	0.872	0.630
	TR2	0.803			
	TR3	0.784			
	TR4	0.812			
Avoidance behavior (AB)	AB1	0.805	0.879	0.877	0.642
	AB2	0.794			
	AB3	0.814			
	AB4	0.790			

**Table 4:** Discriminant validity (FORNELL).

	TR	AB	PS	PSI	PI	PC
TR	0.849					
AB	−0.402	0.856				
PS	0.365	−0.411	0.86			
PSI	0.429	−0.416	0.389	0.852		
PI	0.361	−0.355	0.374	0.4	0.841	
PC	0.41	−0.386	0.388	0.435	0.371	0.844

**Table 5:** Discriminant validity (HTMT).

	TR	AB	PS	PSI	PI	PC
TR						
AB	0.459					
PS	0.416	0.466				
PSI	0.491	0.473	0.443			
PI	0.417	0.406	0.428	0.459		
PC	0.469	0.437	0.442	0.499	0.427	

**Table 6:** VIF values of the inner model matrix.

	TR	AB	PS	PSI	PI	PC
TR		1.398				
AB						
PS	1.324	1.354	1.267			
PSI	1.401	1.473	1.327			
PI	1.32	1.347				
PC	1.372	1.429	1.326			

**Table 7:** Path analysis.

Hypothesis	β	SD	T	*p*	LLCI	ULCI	Decision
H1a.PS→PI	0.209	0.043	4.812	0.000	0.121	0.290	Supported
H1b.PSI→PI	0.237	0.044	5.450	0.000	0.151	0.320	Supported
H1c.PC→PI	0.187	0.044	4.261	0.000	0.100	0.270	Supported
H2a.PS→TR	0.146	0.044	3.330	0.001	0.057	0.230	Supported
H2b.PSI→TR	0.228	0.047	4.864	0.000	0.135	0.318	Supported
H2c.PC→TR	0.202	0.045	4.522	0.000	0.113	0.288	Supported
H3a.PS→AB	−0.194	0.048	4.060	0.000	−0.285	−0.100	Supported
H3b.PSI→AB	−0.170	0.045	3.738	0.000	−0.263	−0.081	Supported
H3c.PC→AB	−0.128	0.046	2.774	0.006	−0.221	−0.04	Supported
H4.PI→TR	0.141	0.043	3.248	0.001	0.056	0.227	Supported
H5.TR→AB	−0.168	0.046	3.681	0.000	−0.256	−0.077	Supported
H6.PI→AB	−0.107	0.045	2.374	0.018	−0.192	−0.017	Supported
H7a.PS→PI→AB	−0.022	0.011	2.020	0.043	−0.048	−0.005	Supported
H7b.PSI→PI→AB	−0.025	0.012	2.177	0.029	−0.051	−0.005	Supported
H7c.PC→PI→AB	−0.020	0.010	2.048	0.041	−0.043	−0.005	Supported
H7d.PS→TR→AB	−0.024	0.010	2.475	0.013	−0.048	−0.008	Supported
H7e.PSI→TR→AB	−0.038	0.013	2.931	0.003	−0.069	−0.017	Supported
H7f.PC→TR→AB	−0.034	0.012	2.731	0.006	−0.064	−0.014	Supported
H7g.PS→PI→TR→AB	−0.005	0.002	2.185	0.029	−0.011	−0.002	Supported
H7h.PSI→PI→TR→AB	−0.006	0.003	2.179	0.029	−0.013	−0.002	Supported
H7i.PC→PI→TR→AB	−0.004	0.002	2.086	0.037	−0.01	−0.002	Supported

**Table 8:** R^2^ values and Q^2^ values.

	R^2^	Q^2^ Prediction
PI	0.242	0.231
TR	0.285	0.259
AB	0.305	0.261

## Data Availability

The data supporting the findings of the current study are available from the corresponding author.
